# Anti-Inflammatory Effect Fraction of *Bletilla striata* and Its Protective Effect on LPS-Induced Acute Lung Injury

**DOI:** 10.1155/2021/6684120

**Published:** 2021-03-13

**Authors:** Chunchun Zhang, Dandan Ning, Jieli Pan, Cheng Chen, Chengxian Gao, Zhishan Ding, Fusheng Jiang, Meiya Li

**Affiliations:** ^1^College of Pharmaceutical Science, Zhejiang Chinese Medical University, Hangzhou 310053, China; ^2^Academy of Chinese Medical Sciences, Zhejiang Chinese Medical University, Hangzhou 310053, China; ^3^College of Life Science, Zhejiang Chinese Medical University, Hangzhou 310053, China; ^4^College of Medical Technology, Zhejiang Chinese Medical University, Hangzhou 310053, China

## Abstract

*Bletilla striata* is a well-known traditional Chinese herb with anti-inflammatory properties that is widely used in the treatment of lung conditions such as silicosis, tuberculosis, and pneumogastric hemorrhage. However, little information on the anti-inflammatory ingredients and their activities is available. In this study, an effect fraction of *Bletilla striata* (EFBS) was enriched, and its anti-inflammatory activities and underlying mechanisms were investigated. EFBS was enriched by polyamide column chromatography and characterized by HPLC; an LPS-induced acute lung injury model was used to evaluate the anti-inflammatory activities of EFBS. Meanwhile, the main anti-inflammation-contributing ingredients and possible molecular mechanism of anti-inflammatory activity in EFBS were verified by component-knockout method combined with LPS-induced RAW264.7 cell model. The EFBS mainly consisted of coelonin (15.88%), batatasin III (32.49%), 3′-*O*-methylbatatasin III (6.96%), and 3-hydroxy-5-methoxy bibenzyl (2.51%). Pretreatment with the EFBS (20 mg/kg and 60 mg/kg) for five days prior to the administration of LPS resulted in decreases in wet-to-dry lung weight ratio, neutrophil number, MPO activity, total protein concentration, NO level, and MDA level, as well as IL-1*β*, IL-6, MCP-1, and TNF-*α* concentrations in the bronchoalveolar lavage fluid. Western blot analysis demonstrated the increased expressions of iNOS, COX-2, and NF-*κ*B p65 in the LPS treatment group, all of which were ameliorated by EFBS pretreatment. Histological examination confirmed the protective effect of the EFBS. Additionally, component-knockout assay confirmed that these four quantitative components contributed significantly to the anti-inflammatory effect of EFBS. Coelonin, batatasin III, 3′-*O*-methylbatatasin III and 3-hydroxy-5-methoxy bibenzyl were the main anti-inflammatory components of EFBS and could regulate the expression of downstream inflammatory cytokines by inhibiting p65 nuclear translocation. These findings uncover, in part, the molecular basis underlying the anti-inflammatory activity of *Bletilla striata*.

## 1. Introduction

*Bletilla striata* (Thunb.) Reichb. f. is a perennial herbaceous orchid known as Baiji (白及) in China. Its dried pseudobulb has been used for millennia in traditional Chinese medicine as an astringent hemostatic agent for the treatment of hemoptysis, hematemesis, traumatic bleeding, and chapped skin [1].

Many conditions can lead to the occurrence of hemoptysis, such as lung cancer [2], tuberculosis [3], and silicosis [4]. Clinical reports have indicated that *Bletilla striata* is beneficial for treating tuberculosis and silicosis [5, 6]. Accordingly, four *Bletilla striata*-related products have been approved by the China Food and Drug Administration (CFDA) [7] for use in the treatment of gastric ulcers and lung-related conditions such as pertussis, tuberculosis, silicosis, chronic tracheitis, and emphysema. However, the active ingredients in these products are largely unknown or not well characterized.

Researchers have attempted to elucidate the pharmacologically active substances in *Bletilla striata* (and their mechanisms of action) from various perspectives. The polysaccharides in *Bletilla striata* are the most widely and deeply studied components, exhibiting wound-healing [8], antiulcer [9], hemostatic [10], and immune modulation activities [11]. However, a growing body of evidence indicates that the small-molecule components of *Bletilla striata* may play more important therapeutic roles. For example, the *n*-butyl alcohol fraction of a 70% ethanol *Bletilla striata* extract was demonstrated to exhibit excellent platelet-aggregation activity [12] while the terpenoid and stilbenoid compounds from *Bletilla striata* were shown to exhibit angiogenesis [13] and antitumor activities [14], respectively. Furthermore, the present authors' previous work has demonstrated that the ethanol extract of *Bletilla striata* effectively prevents silica-induced lung fibrosis by regulating the antioxidation system, the immune system, and cytokine levels [15]. Importantly, this extract was found to be more effective than the polysaccharides in *Bletilla striata* [16]. Clearly, the small-molecule components of *Bletilla striata* are worthy of further research.

Silicosis, an incurable disease, characterized by chronic lung inflammation and progressive fibrosis, affects tens of millions of workers involved in dusty occupations in many countries [17]. Therefore, it is of great significance to explore the pathogenesis of silicosis, develop drugs to prevent, treat, or relieve the symptoms, and improve the quality of life of silicosis patients. Research has indicated that sustained inflammation plays a critical role in silica-induced pulmonary fibrosis [18], and downregulation of inflammation can ameliorate pulmonary fibrosis [19]. Macrophages are key regulators during this process and become the ideal target of anti-inflammatory drug development [20]. The lipopolysaccharide- (LPS-) induced RAW264.7 cell inflammation model *in vitro* and LPS-induced acute lung injury (ALI) model *in vivo* are two accepted methods for investigating the underlying mechanisms of inflammation and screening anti-inflammatory drugs. Based on these models, medicinal plants have been reported to possess numerous secondary metabolites with potent anti-inflammation effects and demonstrated therapeutic promises for inflammation-related diseases [21]. Therefore, medicinal plants are important resources of anti-inflammatory compounds [22]. And our previous studies confirmed that the ethanol extract of *Bletilla striata* has the preventive and therapeutic effect on silica-induced pulmonary fibrosis in rats [15]. We further isolated and identified a series of anti-inflammatory compounds from the ethanol extract of *Bletilla striata* [23, 24], guided by LPS-induced RAW264.7 cell inflammation model, of which coelonin has the strongest anti-inflammatory activity [24, 25]. Meanwhile, an effective fraction of *Bletilla striata* (EFBS) with significant anti-inflammatory activity was prepared [25]. Accordingly, the purpose of this study was to assess the therapeutic efficacy of EFBS in an ALI mouse model, so as to reveal the anti-inflammatory molecular mechanism and provide scientific basis for the establishment of the pharmacodynamic index components of the Chinese herb *Bletilla striata*.

## 2. Materials and Methods

### 2.1. EFBS Preparation

The pseudobulbs of *Bletilla striata* were collected from Meichuan, Wuxue, Hubei province, China, and authenticated by Dr. Zhishan Ding of Zhejiang Chinese Medical University. A voucher specimen (BS No. 20171028) was deposited in the College of Life Science at Zhejiang Chinese Medical University. EFBS were prepared according to our previous study [25]. Briefly, 100.0 g of the tuber powder was reflux extracted in 1 L of 80% ethanol, and this was repeated three times. The filtrate was concentrated under vacuum to 600 mL, and an equal volume of distilled water along with 30.0 g polyamide (100-200 mesh, Taizhou Luqiao Sijia Biochemical Plastics Factory, Zhejiang, China) was added. The suspension was then further concentrated under vacuum to 600 mL. Finally, the suspension was packed into a suitable column and successively eluted with water, 20% ethanol, and 40% ethanol, and the 40% ethanol elution was collected, concentrated, and dried under vacuum to obtain the EFBS under investigation. The EFBS was characterized, and the main component contents were obtained by HPLC. Briefly, each sample was injected (10 *μ*L) and analyzed using a Dionex Ultimate™ 3000 HPLC system (Dionex, California, USA) with pulsed amperometric *detection* at 193 nm. An Acclaim® 120 C18 (25 × 4.6 mm, 5 *μ*m) HPLC column protected with a Phenomenex security guard column (C18, 4 × 3.0 mm) operated at 30°C was used, and the flow rate was maintained at 1 mL/min. The elution solvents were acetonitrile (A) and 0.1% acetic acid (B). Samples were eluted according to the following gradient: 0-5 min 10% to 40% A, 5-12 min 40% A isocratic, 12-16 min 40% to 45% A, 16-22 min 45% A isocratic, 22-25 min 45% to 48% A, and 25-33 min 48% A isocratic, and finally washing and reconditioning of the column.

### 2.2. The Four Main Compounds Knockout-EFBS (KO-EFBS) Preparation

To better reveal the contribution of four main components to the anti-inflammatory activity of EFBS, the four compounds were removed by semipreparative liquid chromatography on a Dionex Ultimate™ 3000 semiprepared HPLC system (Dionex, California, USA) to obtain sample KO-EFBS. A Welch Ultimate® XB-C18 (10 × 250 mm, 10 *μ*m) HPLC column operated at 30°C was used, and the flow rate was maintained at 5 mL/min, and *detection* at 193 nm. Samples were eluted with acetonitrile (A) and 0.1% acetic acid (B) as the following gradient: 0-26 min 32% A isocratic, 26-28 min 32%-55% A, 28-38 min 55%A isocratic, 38-39 min 55%-90% A, 39-43 min 90% A isocratic, 43-44 min 90%-32% A, and 44-54 min 32% A isocratic. And meanwhile, the four main component peaks were also collected and mixed to prepare mixed-compounds 1-4 fraction (MC1-4). Both KO-EFBS and MC1-4 were characterized by the HPLC method described above.

### 2.3. Animals and Cell Culture

Male Institute of Cancer Research (ICR) mice were obtained from the SIPPR/BK Laboratory Animal Company (Shanghai, China) and were used at 6–8 weeks of age (20-25 g). The mice were caged under pathogen-free conditions (22 ± 3°C, 12 h light/12 h darkness cycle, 45-55% relative humidity) and allowed free access to water and food during the study period. All experiments were performed in full compliance with standard laboratory animal care protocols approved by the Institutional Animal Care Committee of Zhejiang Chinese Medical University.

RAW264.7 cells (ATCC) were purchased from the Type Culture Collection of the Chinese Academy of Sciences, Shanghai, China. The cells were maintained in DMEM medium (Gibco, USA) containing 10% heat inactivated fetal bovine serum (Gibco, USA), 100 units/mL penicillin, and 100 *μ*g/mL streptomycin. They were cultured at 37°C in a humidified atmosphere of 5% CO_2_ incubator.

### 2.4. Drug and LPS Administration

The mice were randomly divided into five groups (control group, model group, DEX (5 mg/kg, Sigma) group, EFBS (20 mg/kg) group, and EFBS (60 mg/kg) group). The control and model groups were administrated equal volumes of vehicle orally, and the DEX and EFBS groups were administered intraperitoneally and orally, respectively. All groups were administered continuously for five days, and 5 mg/kg LPS was infused 1 h after the last administration. Mice were anesthetized by intraperitoneal injection of pentobarbital (50 mg/kg, Sigma), and then LPS (5 mg/kg, Sigma) was intratracheally instillated. The control group was administered an equal volume of PBS. All the mice were sacrificed 6 h after LPS administration.

### 2.5. Lung Wet-to-Dry Weight Ratio

Lung edema was calculated by reference to the *lung wet-to-dry weight ratio* (W/D). The right lung was ligated and excised, and the wet weight of the upper lobes of right lung was determined. The lungs were then placed in an incubator at 60°C for three days to obtain the dry weight, and the W/D weight ratio was then calculated.

### 2.6. Bronchoalveolar Lavage Fluid Collection and Cell Count

Following the removal of the right lung, the bronchoalveolar lavage fluid (BALF) was collected by lavaging with 3 × 0.5 mL of PBS containing 0.1 mM EDTA. Then the BALF samples were centrifuged at 600 × g for 10 min at 4°C, and the supernatants were collected for cytokine determination. The cell pellets were resuspended in 1 mL of PBS to count the total cells and neutrophils using a hemocytometer and staining by the Wright-Giemsa method.

### 2.7. Assessment of Capillary Leakage

Inflammation factors dramatically increase the permeability of alveolar-capillary barriers and thus following inflammation cell and protein infiltration. Hence, the total protein concentration in the BALF supernatant was determined using a bicinchoninic acid (BCA) protein assay kit (Beyotime Biotechnology, China).

### 2.8. Measurement of MPO Activity and Oxidant Stress

As an index of neutrophil infiltration, the BALF MPO activity was measured with assay kits (Multi Sciences Biotechnology, Shanghai, China) according to the manufacturer's instructions.

The concentrations of NO and MDA in the BALF supernatant were determined using commercially available colorimetric assay kits (Beyotime Biotechnology, China) according to the manufacturer's instructions.

### 2.9. Measurement of BALF Cytokine Levels

Levels of IL-1*β*, TNF-*α*, IL-6, and MCP-1 in the BALF were quantified using the CBA method according to the manufacturer's protocols (BD, USA) using a BD Accuri™ C6 flow cytometer (BD, USA).

### 2.10. Lung Histopathology

The lower lobes of right lung were fixed with 10% neutral formalin overnight at room temperature, and specimens were dehydrated in graded alcohol and embedded in paraffin. Then, 4 *μ*m-thick paraffin sections were obtained and stained with hematoxylin and eosin (H&E) according to the standard protocol. The degree of pathological injury was scored based on neutrophils infiltration, edema, hemorrhage, and disorganization of lung parenchyma, as the scoring system described by Kiyonari et al. [26]. The damage degree of each index was scored with values from 0 to 4, and higher scores suggest more severe damage presented. Finally, total histological scores were calculated [27].

### 2.11. *In Vitro* Cell Viability Assay

RAW264.7 cells were seeded into 96-well plates at a density of 5 × 10^4^ cells/well and were treated with different concentrations of EFBS for 48 h. Then, cell viability was determined using CCK8 assay according to the manufacturer's instructions. Briefly, 10 *μ*L CCK8 reagent (Dojindo, Tokyo, Japan) was added to each well and incubated for 3 hours at 37°C. Absorbance was measured at 450 nm on a microplate reader.

### 2.12. Cell Apoptosis Assay

RAW264.7 cells were seeded into 6-well plates at a density of 1 × 10^6^ cells/well and were treated with increasing concentrations of EFBS for 48 h. Cells were detected using the Annexin V-FITC apoptosis detection kit (556547, BD Pharmingen, USA) and analyzed on a Beckman CytoFLEX S flow cytometer (Beckman Coulter, CA.USA).

### 2.13. Measurement of Cell Culture Supernatant Cytokine Levels

RAW264.7 cells were pretreated with EFBS, KO-EFBS, MC1-4, or KO-EFBS combined with MC1-4 for 1 h and followed by stimulation with LPS (200 ng/mL) for 12 h. The culture supernatant was collected for IL-6 and TNF-*α* detection. The remaining cells were then treated by 1 mM ATP for additional 15 min at 37°C [28]; then, supernatants were collected for IL-1*β* detection. All cytokines were quantified using the Cytometric Beads Array (CBA) method according to the manufacturer's protocols (BD, USA).

### 2.14. Western Blot Analysis

The middle lobes of right lung tissue were washed twice in ice-cold PBS, cut into small pieces, and homogenized in cold protein lysis buffer (Thermo Fisher Scientific, USA) with protease and phosphatase inhibitors (Roche, Germany). Total proteins were obtained by incubating the lysates at 4°C for 30 min with vortex mixing, followed by centrifugation at 14,000 × g for 15 min at 4°C, and the supernatant was transferred to a new tube for analysis.

For cell culture samples, RAW264.7 cells were pretreated by EFBS, KO-EFBS, MC1-4, KO-EFBS combined with MC1-4, or single compounds for 1 h and then stimulated with LPS (200 ng/mL) for 30 min or 24 h. Cells were collected, and whole-cell proteins were extracted with M-PER Mammalian Protein Extraction Reagent (78503, Thermo Fisher Scientific, USA) at 4°C for 30 min. Then, the samples were centrifuged at 14,000 × g for 15 min, and the supernatant was transferred to a new tube to analyze the expression of iNOS and COX2 (24 h treated samples) and the phosphorylation of JNK, ERK1/2, and p38 (30 min treated samples). And nuclear proteins were extracted in accordance with the instruction of the Nuclear and Cytoplasmic Protein Extraction Kit (P0027, Beyotime Biotechnology, China) for the detection of the nuclear translocation of p65.

Before blotting, the protein was quantified using the BCA method. Simple western immunoblotting was performed on a Simple Wes system (ProteinSimple, California, USA) using a Size Separation Master Kit with Split Buffer (12-230 kDa) according to the manufacturer's standard instruction and using anti-iNOS (EPR16635) (ab178945, Abcam, USA), anti-NF-*κ*B p65 (E379) (ab32536, Abcam, USA), anti-COX2 (EPR12012) (ab179800, Abcam, USA), anti-p38 (ab170099, Abcam, USA), anti-p38(phospho T180+Y182) (ab195049, Abcam, USA), anti-JNK1+JNK2+JNK3 (ab208035, Abcam, USA), anti-JNK1+JNK2+JNK3(phospho T183+T183+T221) (ab124956, Abcam, USA), anti-ERK1+ERK2 (ab54230, Abcam, USA), anti-Erk1(pT202/pY204)+Erk2 (pT185/pY187) (ab50011, Abcam, USA), anti-Lamin A/C antibody (2032S, CST, USA), and anti-*β*-actin (4970S, CST, USA) antibodies. The Compass software (version 4.0.0, ProteinSimple) was used to program the Simple Wes and for presentation (and quantification) of the western immunoblots. Output data were displayed from the software as calculated averages of seven exposures (5-480 s).

### 2.15. Statistical Analysis

All data are presented as mean ± SD, and eight animals are included in each group. Statistical analysis was carried out using Graph Pad Prism 6.0 (La Jolla, CA) and performed using a one-way ANOVA followed by Dunnett's test. *P* < 0.05 is accepted as the level of significance.

## 3. Results

### 3.1. Characterization and Quantification of EFBS

**Figure 1:**
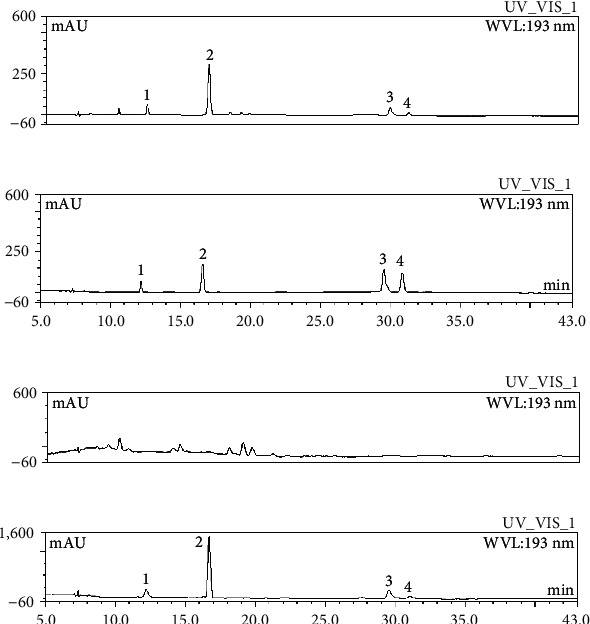
HPLC characterization of the EFBS (a). Standards were prepared in our laboratory, with purity greater than 98% (b). KO-EFBS (c). MC1-4 (d). 1–4 represent coelonin, batatasin III, 3′-*O*-methylbatatasin III, and 3-hydroxy-5-methoxy bibenzyl, respectively.

The EFBS was prepared and characterized by HPLC. It is mainly composed of stilbenes [6, 29] and the compounds we previously identified [23]. Quantitative analysis of the four main components coelonin, batatasin III, 3′-*O*-methylbatatasin III, and 3-hydroxy-5-methoxy bibenzyl [24] in EFBS was performed, and their contents were revealed to be 15.88%, 32.49%, 6.96%, and 2.51%, respectively (Figure 1(a)). A standardized EFBS sample (Figure 1(b)) was used for subsequent pharmacological studies.

Moreover, to investigate the potential function and anti-inflammatory contribution of the four main compounds to EFBS, we prepared the four compound-free extract from the EFBS (KO-EFBS) and the four compound-mixed fraction (MC1-4) using semipreparative liquid chromatography. As shown in Figure 1(c), the corresponding retention time peaks of four compounds in EFBS disappeared, which were mainly enriched in MC1-4 (Figure 1(d).

### 3.2. Effects of EFBS on the W/D Ratio

**Figure 2:**
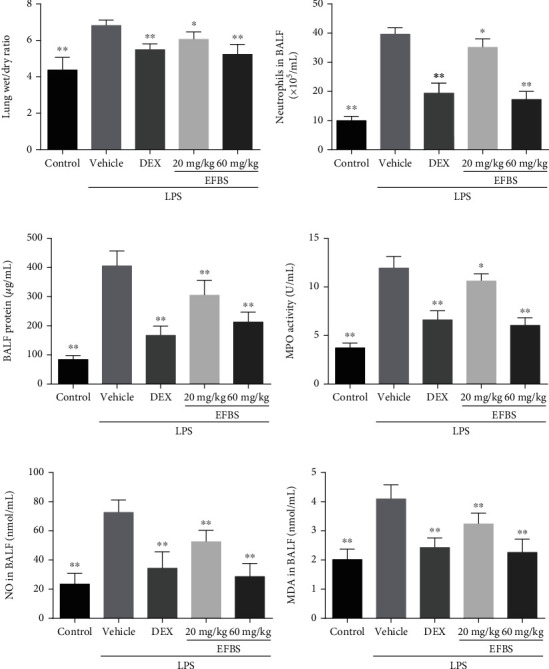
Lung W/D weight ratios for different groups (a). Protein concentration (b), neutrophil numbers (c), MPO activity (d), NO concentration (e), and MDA concentration (f) in BALF. Data are expressed as mean ± SD (*n* = 8). ∗*P* < 0.05, ∗∗*P* < 0.01 compared with the vehicle treatment group.

The W/D ratio was used to evaluate LPS-induced pulmonary vascular permeability to water. As shown in Figure 2(a), LPS administration causes a significant increase in lung W/D weight ratio compared with the control. Both EFBS and DEX significantly decrease the magnitude of pulmonary edema, and the high-dose EFBS treatment group (60 mg/kg) exhibits a comparable effect to that of DEX (5 mg/kg).

### 3.3. Effects of EFBS on Protein and Neutrophil Infiltration

The lung W/D weight ratio increase induced by LPS is mainly due to alveolar-capillary barrier dysfunction, resulting in infiltration of water, proteins, and inflammatory cells. Thus, the protein concentration and neutrophil numbers in BALF were determined. As shown in Figure 2, LPS treatment dramatically increases the protein concentration (Figure 2(b)) and neutrophil number (Figure 2(c)) in BALF. However, pretreatment with DEX or EFBS significantly ameliorates the effect of LPS.

### 3.4. Effects of EFBS on MPO Activity

LPS recruits a large number of neutrophils into lung tissue and causes significant release of MPO, inflammatory cytokines, and reactive oxygen species (ROSs), leading to lung tissue damage. Hence, the MPO activity in BALF was detected to assess the effect of EFBS on neutrophil accumulation. As shown in Figure 2(d), LPS treatment causes significant increase MPO activity in BALF, and this is lessened by both low- and high-dosage EFBS pretreatment. This implies that EFBS pretreatment ameliorates neutrophil accumulation in lung tissue.

### 3.5. Effect of EFBS on Oxidative Stress

ROSs such as NO are produced by a variety of cell types and can cause oxidative stress and directly damage lung tissue. As expected, NO levels are significantly increased by LPS stimulation, and this increase is dramatically reduced by both DEX and EFBS preadministration (Figure 2(e)). High levels of ROSs can lead to lipid peroxidation injury, and the levels of MDA, a lipid peroxidation product, indirectly reflect the extent of such injury. As shown in Figure 2(f), the changes in MDA level follow the same pattern as those of NO. Furthermore, it is noteworthy that the EFBS (60 mg/kg) treatment group exhibits a comparable effect to that of the DEX (5 mg/kg) group.

### 3.6. Effects of EFBS on the Concentration of Cytokines in BALF

**Figure 3:**
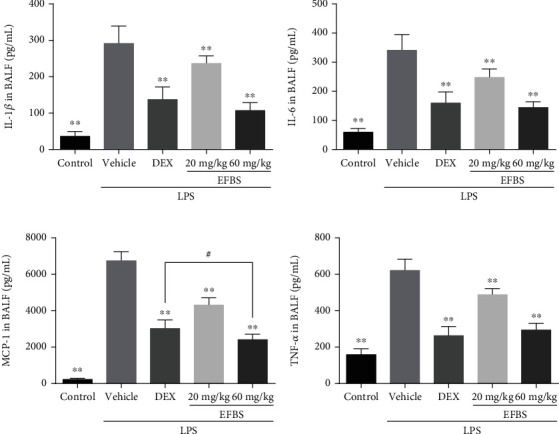
Concentrations of proinflammatory cytokines in BALF. Data are expressed as mean ± SD (*n* = 8). ∗∗*P* < 0.01 compared with the vehicle treatment group.

The concentrations of the proinflammatory cytokines IL-1*β*, IL-6, MCP-1, and TNF-*α* in BALF were determined. These cytokines activate and recruit neutrophils into the lung tissue. The levels of all four cytokines are dramatically elevated by LPS challenging. However, both low and high dosage of EFBS intake reduces the levels of all four markers (Figure 3). Additionally, EFBS (60 mg/kg) exerts greater inhibitory activity than DEX (5 mg/kg) on MCP-1.

### 3.7. Effects of EFBS on Lung Tissue Damage

**Figure 4:**
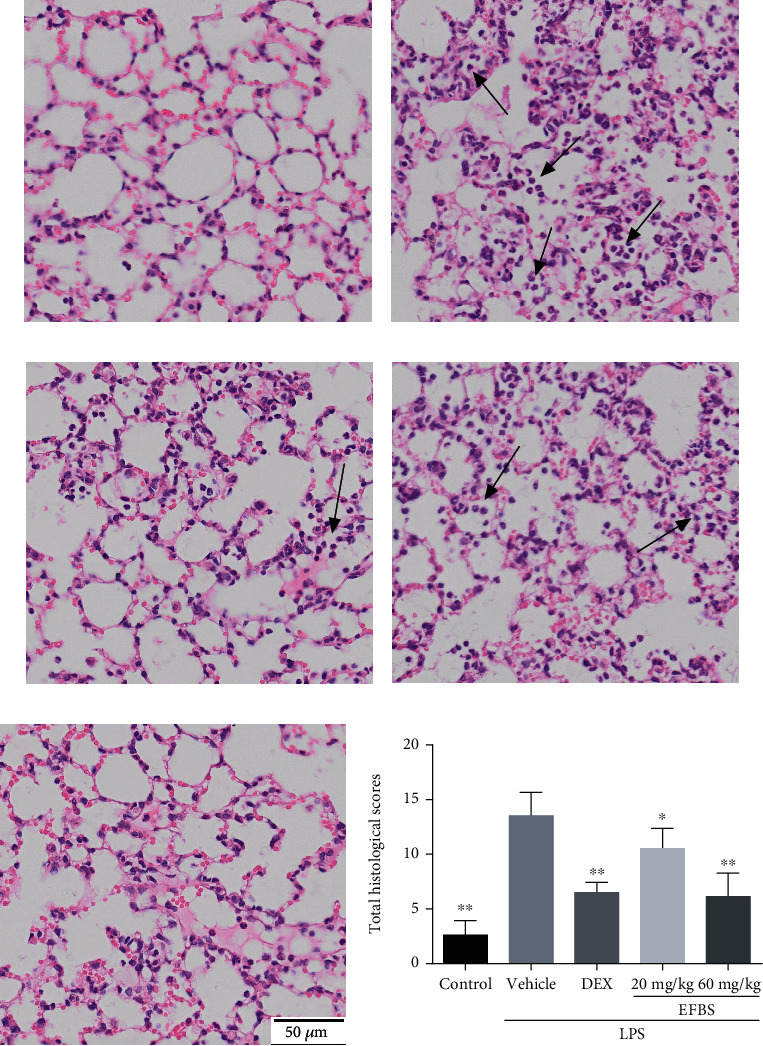
The effects of EFBS on changes to lung histopathology (a–e) and total histopathological scores (f) induced by LPS. The ICR mice were (a) left untreated or challenged with LPS (5 mg/kg) for 6 h following (b) no pretreatment or pretreatment with (c) 5 mg/kg DEX, (d) 20 mg/kg EFBS, or (e) 60 mg/kg EFBS. Black arrow indicates neutrophils infiltration.

The effects of EFBS on LPS-induced lung histopathologic changes were assessed by H&E staining. As illustrated in Figure 4, the untreated control group shows normal morphological lung tissue structure. Conversely, increased thickness of the alveolar wall, massive neutrophil infiltration, and the destruction of alveolar structure are observed in the LPS-treated group. However, the total lung injury scores were dramatically reduced both by pretreatment with low- and high-dosage EFBS (Figure 4(f)).

### 3.8. Effects of EFBS on Lung Tissue COX2, iNOS, and NF-*κ*B Expression

**Figure 5:**
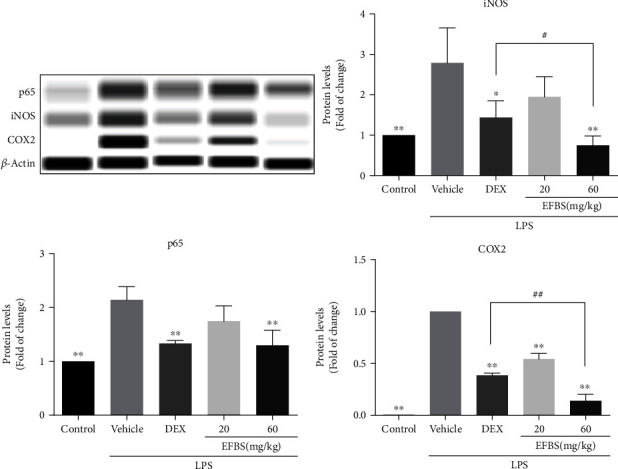
Effect of EFBS on LPS-induced COX2, iNOS, and NF-*κ*B p65 expression in lung tissue. The ICR mice were left untreated or challenged with LPS (5 mg/kg) for 6 h following no pretreatment or preadministration of 5 mg/kg DEX, 20 mg/kg EFBS, or 60 mg/kg EFBS. All mice were sacrificed 6 h after LPS administration, and lung tissue was collected for western blot analysis with specific antibodies. Data are expressed as mean ± SD (*n* = 3). ∗*P* < 0.05, ∗∗*P* < 0.01 compared with the vehicle treatment group. ^#^*P* < 0.05, ^##^*P* < 0.01 compared with the DEX treatment group.

The expression of the inflammation-related genes COX2, iNOS, and NF-*κ*B p65 in the lung tissue was measured by western blotting, revealing that LPS challenge significantly elevates COX2, iNOS, and NF-*κ*B p65 expression as compared to the control group. As expected, pretreatment with low- and high-dosage EFBS both effectively decreases the levels of COX2, iNOS, and NF-*κ*B p65. The levels of these three proteins are also significantly inhibited by DEX preadministration compared to the vehicle treated group (Figure 5). Surprisingly, the inhibition of iNOS and COX-2 in high-dosage EFBS treatment group was significantly stronger than those in DEX treatment group.

### 3.9. Effects of EFBS on RAW264.7 Cell Viability and Cytokines Secretion

**Figure 6:**
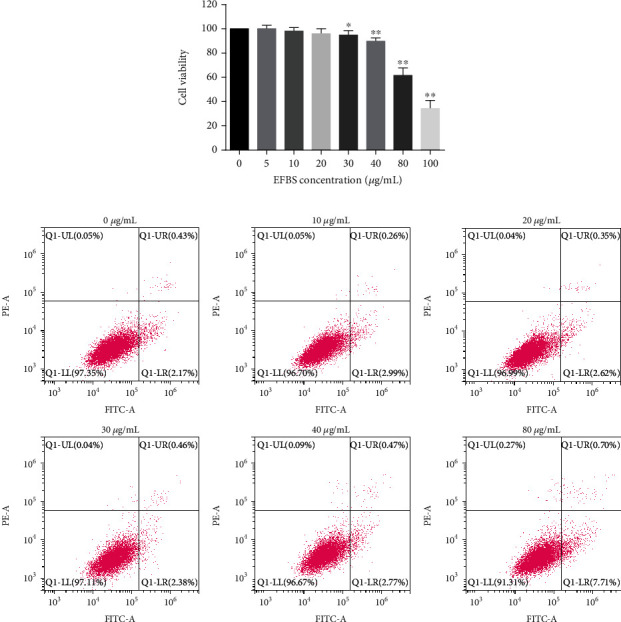
Cell viability (a) and cell apoptosis (b) treated by different concentrations of EFBS. Statistical analysis was performed using one-way ANOVA followed by the Tukey-Kramer posttest. ∗*P* < 0.05, ∗∗*P* < 0.01 compared with vehicle treatment group.

Cytotoxicity of EFBS on RAW264.7 cells was determined by the CCK8 assay. Cells were treated with various concentrations of EFBS for 48 h. As depicted in Figure 6(a), at the concentrations of 40 *μ*g/mL, 80 *μ*g/mL, and 100 *μ*g/mL, EFBS remarkably decreased RAW264.7 cells viability, while cells viability was not greatly impacted by EFBS at concentrations below 30 *μ*g/mL. However, flow cytometry analysis showed that EFBS did not induce cell apoptosis and death at the concentration of 40 *μ*g/mL, but significant cells apoptosis was observed at the concentration of 80 *μ*g/mL (Figure 6(b)). These results indicate that EFBS has no obvious direct cytotoxicity below 40 *μ*g/mL but can significantly inhibit the proliferation of RAW264.7 cells at 40 *μ*g/mL.

**Figure 7:**
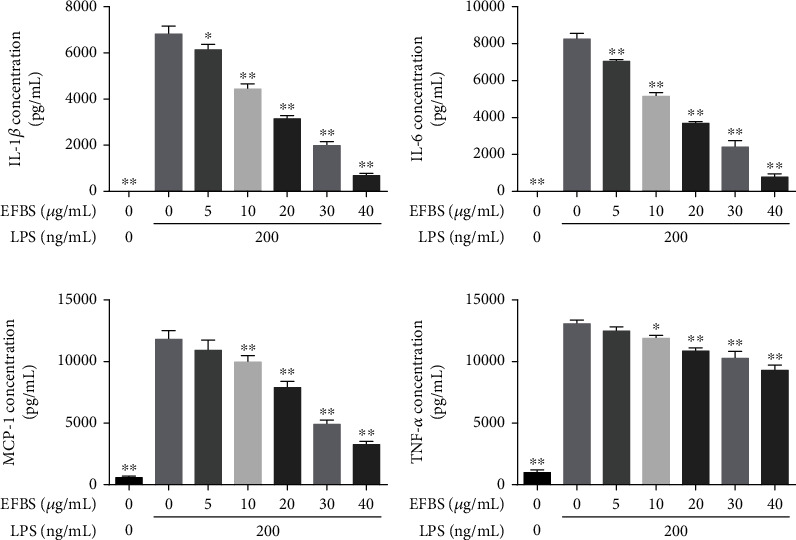
Proinflammatory cytokines level treated by different concentrations of EFBS. Statistical analysis was performed using one-way ANOVA followed by the Tukey-Kramer posttest. ∗*P* < 0.05, ∗∗*P* < 0.01 compared with the LPS treatment group.

To further assess the anti-inflammation activity of EFBS, RAW264.7 cells were pretreated with different concentrations of EFBS for 1 hour, then challenged with 200 ng/mL LPS for 12 hours, and the cell culture supernatant was collected to determine the levels of cytokines. As shown in Figure 7, LPS-induced upregulation of cytokine levels of IL-1*β*, IL-6, MCP-1, and TNF-*α* was markedly inhibited by the treatments with EFBS in a dose-dependent manner. When EFBS was treated at 30 *μ*g/mL, the inhibition ratio of IL-1*β*, IL-6, MCP-1, and TNF-*α* reached 70.74%, 71.04%, 58.28%, and 21.21%, respectively. Therefore, EFBS in a concentration of 30 *μ*g/mL was used in the subsequent studies.

### 3.10. Effects of EFBS on Expression of COX2 and iNOS and Phosphorylation of JNK, ERK1/2, and p38 in LPS-Induced RAW264.7 Cells

**Figure 8:**
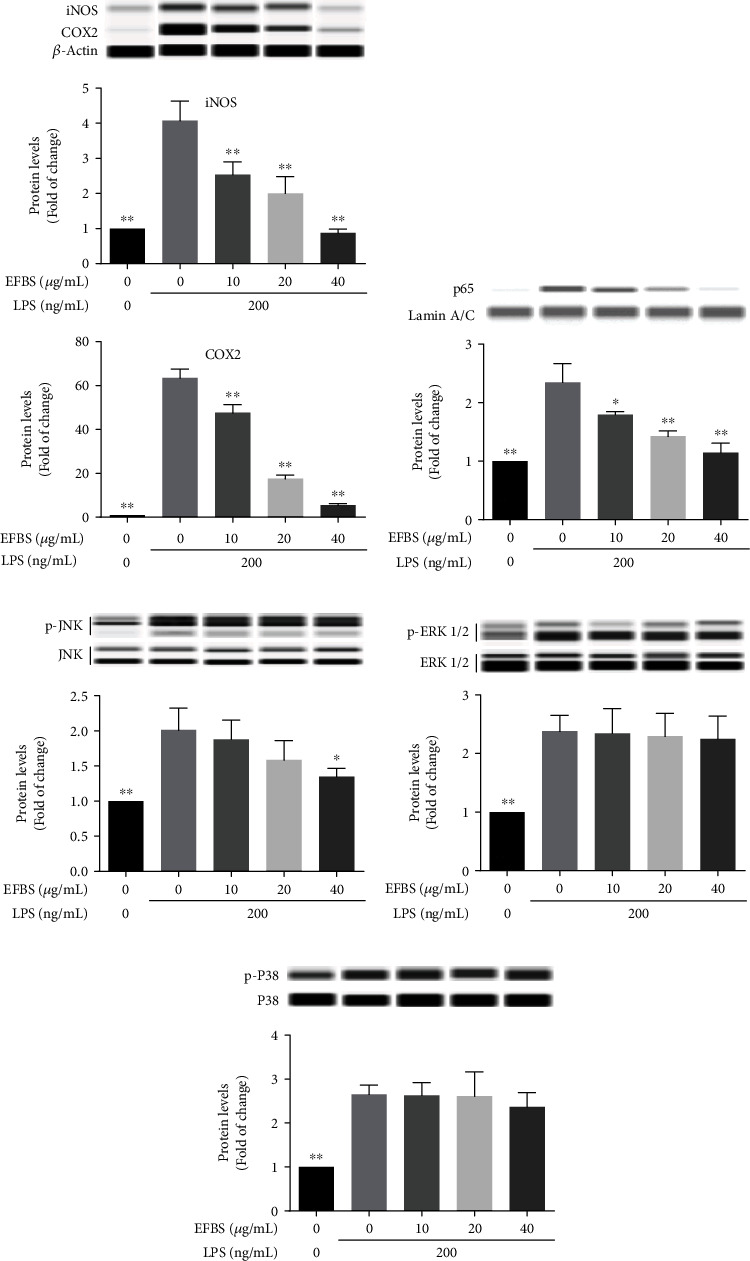
Regulation of EFBS on NF-*κ*B and MAPK signaling pathway and its inhibitory effect on iNOS and COX2 expression. Statistical analysis was performed using one-way ANOVA followed by the Tukey-Kramer posttest. ∗*P* < 0.05, ∗∗*P* < 0.01 compared with the LPS treatment group.

MAPK signaling pathway is also involved in the regulation of proinflammatory gene transcription [30]. As shown in Figure 8, except the inhibition of 40 *μ*g/mL of EFBS on JNK phosphorylation (Figure 8(c)), EFBS pretreatment was observed no noticeable changes in the phosphorylation of JNK, ERK1/2, and p38 induced by LPS. As expected, similar to the results *in vivo*, EFBS could also dramatically inhibit the expression of iNOS and COX-2, as well as prevention of NF-*κ*B p65 nuclear translocation (Figure 8(a)) in a dose-dependent manner on LPS-induced RAW264.7 cells. These results implied that EFBS plays an anti-inflammatory role mainly through regulating the NF-*κ*B pathway.

### 3.11. Effect of KO-EFBS and Combination of KO-EFBS and MC1-4 on LPS-Induced Cytokines Secretion

**Figure 9:**
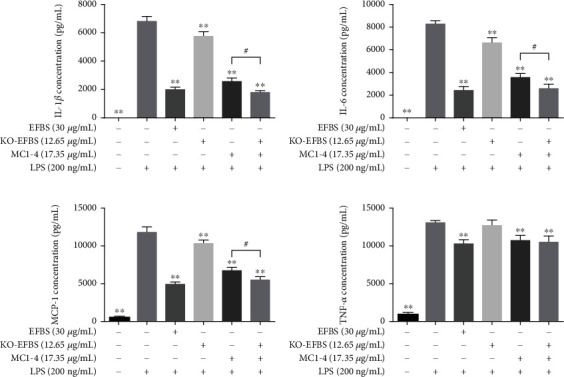
Proinflammatory cytokines inhibitory effect of KO-EFBS and MC1-4. Statistical analysis was performed using one-way ANOVA followed by the Tukey-Kramer posttest. ∗∗*P* < 0.01 compared with LPS treatment group. ^#^*P* < 0.05.

We evaluated the inhibitory effect of KO-EFBS and the combined treatment with MC1-4 on LPS-induced cytokine secretion. Since 30 *μ*g of EFBS contains 17.35 *μ*g of MC1-4 and 12.65 *μ*g KO-EFBS, RAW264.7 cells were pretreated with EFBS (30 *μ*g/mL), KO-EFBS (12.65 *μ*g/mL), MC1-4 (17.35 *μ*g/mL), or the combination of KO-EFBS (12.65 *μ*g/mL) and MC1-4 (17.35 *μ*g/mL). Results indicated that the treatment of EFBS dramatically suppressed IL-1*β*, IL-6, MCP-1, and TNF-*α* expression as compared to LPS treatment (Figures 9(a)–9(c)). However, the inhibitory activity of KO-EFBS was significantly lower than that of EFBS, although the inhibitory effect of KO-EFBS on other cytokines except TNF-*α* is still with statistical significance vs. LPS. On the contrary, MC1-4 showed EFBS-like inhibitory activity, and the combined treatment with KO-EFBS improved the inhibitory activity against IL-1*β*, IL-6, and MCP-1 (Figures 9(a)–9(c)). These results implied that MC1-4 plays an important role in the anti-inflammatory activity of EFBS.

### 3.12. Effect of the Four Compounds on LPS-Induced Cytokines Secretion

**Figure 10:**
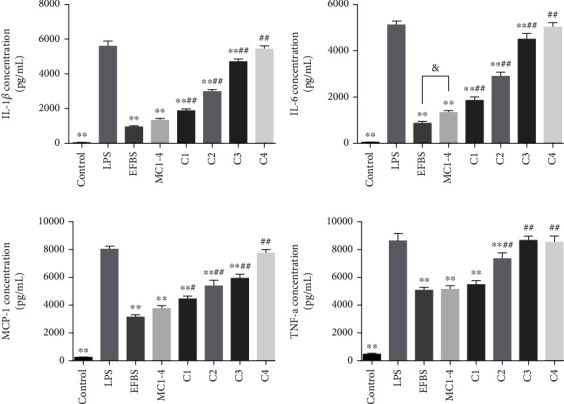
Proinflammatory cytokines inhibitory effect of the four compounds. RAW264.7 cells were pretreated with EFBS (30 *μ*g/mL), MC1-4 (17.35 *μ*g/mL), C1(4.76 *μ*g/mL), C2 (9.75 *μ*g/mL), C3 (2.09 *μ*g/mL), or C4 (0.75 *μ*g/mL) for 1 h and followed by stimulation with LPS (200 ng/mL) for 12 h. The culture supernatant was collected for IL-6, MCP-1, and TNF-*α* detection. The remaining cells were then treated by 1 mM ATP for additional 15 min at 37°C; then, supernatants were collected for IL-1*β* detection. All cytokines were quantified using the Cytometric Beads Array (CBA) method. Statistical analysis was performed using one-way ANOVA followed by the Tukey-Kramer posttest. ∗∗*P* < 0.01 compared with the LPS treatment group. ^#^*P* < 0.05, ^##^*P* < 0.01 compared with the MC1-4 treatment group. ^&^*P* < 0.05.

In order to further reveal the contribution of anti-inflammatory activities of the four compounds, coelonin, batatasin III, 3′-*O*-methylbatatasin III, and 3-hydroxy-5-methoxy bibenzyl, in MC1-4 and EFBS, the anti-inflammatory activities of each compound were analyzed according to its proportion in EFBS. As depicted in Figure 10, MC1-4 (17.35 *μ*g/mL) still manifested most of the anti-inflammatory activity of EFBS (30 *μ*g/mL). However, compound 3-hydroxy-5-methoxy bibenzyl (C4) showed no significant inhibitory effect on all inflammatory cytokines at concentration 0.75 *μ*g/mL. And 3′-*O*-methylbatatasin III (C3) also showed no inhibitory activity on TNF-*α* at concentration 2.09 *μ*g/mL, but it could statistically downregulate IL-1*β*, IL-6, and MCP-1 secretion. It was noteworthy that both compounds coelonin (C1, 4.76 *μ*g/mL) and batatasin III (C2, 9.75 *μ*g/mL) had remarkably inhibitory effects on all cytokines, in particular, the former demonstrated the same TNF-*α* inhibition rate as MC1-4 (Figure 10(d)). Additionally, the inhibitory activities of the four compounds on IL-1*β*, IL-6, and MCP-1 were significantly lower than those of their mixture, MC1-4, which indicated that there might be synergistic interaction between these compounds, which increased the anti-inflammatory activity. The exact interaction molecular mechanism is worth further study.

### 3.13. Effect of KO-EFBS and Combination of KO-EFBS and MC1-4 on LPS-Induced p65 Nuclear Translocation

**Figure 11:**
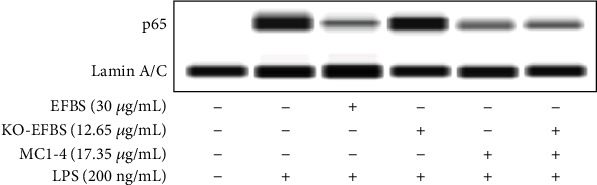
Western blot of p65 nuclear translocation.

NF-*κ*B plays a pivotal role in LPS-induced macrophage activation and inflammatory response. While EFBS mainly inhibits the expression of inflammatory cytokines through the inactivation of NF-*κ*B, therefore, in order to verify the role of MC1-4 in the inhibition of NF-*κ*B activity by EFBS, we conducted western blotting. As shown in Figure 11, LPS challenge significantly induced p65 nuclear translocation, while this effect was dramatically inhibited by EFBS, MC1-4, or combination of KO-EFBS and MC1-4 pretreatment. However, KO-EFBS vs. LPS treatment showed no significant attenuation effect. Moreover, we had confirmed that coelonin and batatasin III also play an anti-inflammatory role mainly by inhibiting NF-*κ*B activation [24]. This further suggests that the regulation of NF-*κ*B pathway is one of the main molecular mechanisms of the anti-inflammatory activity of EFBS.

## 4. Discussion

LPS is a major component derived from the outer membranes of Gram-negative bacteria that causes endothelial barrier damage and subsequently recruitment of inflammatory cells into the lung, resulting in ALI [31]. This phenomenon can be reproduced in many different animal species, and the main symptoms are similar to those of human ALI and acute respiratory distress syndrome (ARDS). Thus, ALI models provide a very useful method for discovering new therapeutic targets and developing new drugs for ALI and ARDS treatment. In the current study, an LPS-induced ALI mouse model was used to study the preventive effects and underlying mechanisms of the EFBS from *Bletilla striata*.

LPS directly induces pulmonary endothelial barrier dysfunction by increasing endothelial cell skeleton contraction and indirectly induces pulmonary endothelial barrier dysfunction by activating neutrophils, macrophages, and other cells to produce oxidative stress, cytokine secretion, and inflammatory reaction [32]. Endothelial barrier dysfunction results in increased permeability, protein and inflammation cell infiltration, and lung edema [33]. Accordingly, we found that LPS challenge significantly increases lung W/D ratio, protein concentration, and total cell numbers in BALF. However, EFBS not only remarkably reduces W/D weight ratio, protein concentration, and total cells in BALF but also greatly improves the microstructure of lung tissue and reduces neutrophil infiltration in ALI. The results confirmed that EFBS has a beneficial effect on the ALI induced by LPS. However, neutrophil infiltration into the tissue is heterogeneous [34], so histopathological results cannot effectively show the overall invasion of neutrophils. Conversely, the activity of MPO, an enzyme mainly secreted by neutrophils [35], can quantitatively reflect the severity of neutrophil invasion of lung tissue. Our results indicated that EFBS dose-dependently reduces MPO activity as induced by LPS in BALF, which is consistent with the number of cells in BALF and the infiltration of neutrophils found by histopathology.

Excessive and persistent accumulation of neutrophils can cause injury to the lung by secreting ROSs, inflammatory cytokines, and proteases [35]. Excess ROSs can induce lipid peroxidation to produce MDA and even lead to cell death, which activates macrophages, neutrophils, and endothelial cells. These activated cells further produce free radicals, cytokines, and chemokines, which amplify the signal cascade and ultimately lead to lung injury [36]. Thus, we measured the NO, MDA, TNF-*α*, IL-1*β*, IL-6, and MCP-1 levels in BALF. As expected, EFBS pretreatment significantly decreases the levels of the above mediators, indicating that EFBS has protective effects against LPS-induced ALI, probably due to its ROS-scavenging and inflammatory-cytokine-inhibition activities.

Inducible nitric oxide synthase (iNOS), which is responsible for over production of NO and leads to lung tissue damage via peroxynitrite formation [37], is significantly increased in both acute respiratory distress syndrome patients [38] and ALI animal models [39]. Since EFBS inhibits NO levels in a dose-dependent manner, we speculate that EFBS may act by inhibiting iNOS. Western blot results confirmed that EFBS dose-dependently inhibits iNOS expression.

Like iNOS, COX2, an inducible isoform of cyclooxygenase (COX), increases significantly upon lung injury and further aggravates the inflammatory response, leading to lung tissue damage [40]. As expected, EFBS pretreatment significantly reduces the levels of COX2. Thus, we propose that the protective effects of EFBS against LPS-induced ALI may be partially due to downregulation of COX2 and iNOS expression. Moreover, it is well known that NF-*κ*B is a key regulator of proinflammatory mediators, regulating the expression of many proinflammatory genes such as TNF-*α*, IL-1*β*, IL-6, iNOS, and COX2 [41]. Western blot analysis indicated that EFBS significantly downregulates NF-*κ*B p65 expression in a dose-dependent manner. Thus, downregulation of NF-*κ*B activity and blocking of its signal transduction pathway may be one of the major anti-inflammatory mechanisms of EFBS in LPS-induced ALI.

Since it has been well documented that MAPKs signal pathway was also participated in regulating proinflammatory cytokines secretion [42], phosphorylation inhibitory effect of EFBS on JNK, ERK1/2 and p38 was also studied. And the results indicated that only 40 *μ*g/mL of EFBS has a statistic inhibitory effect on JNK phosphorylation, while LPS-induced accumulation of NF-*κ*B p65 in nuclear was effectively inhibited by EFBS in the range of 10-40 *μ*g/mL. Obviously, both *in vitro* and *in vivo* data supported that EFBS may play an anti-inflammatory role mainly by inhibiting NF-*κ*B pathway.

To further clarify the importance and representativeness of the four compounds used to standardize EFBS in the anti-inflammatory process of EFBS; we performed component-knockout technique combined with LPS-induced RAW264.7 cell inflammation model. The results showed that EFBS could inhibit the expression of LPS-induced inflammatory cytokines in a dose-dependent manner, which was consistent with the results of ALI model. However, the anti-inflammatory activity of KO-EFBS decreased significantly after the removal of four components, but MC1-4, a mixture of four components, showed strong anti-inflammatory activity. In addition, both MC1-4 and EFBS play a regulatory role in the expression of downstream inflammatory cytokines by inhibiting p65 nuclear translocation, while the role of KO-EFBS is weakened. Therefore, these four components are the main material basis of EFBS, and inhibition of activation of NF-*κ*B may be one of the molecular mechanisms for EFBS to exert its anti-inflammatory activity.

Furthermore, the inhibitory activities of the four monomers at the corresponding concentration in 30 *μ*g/mL EFBS were also analyzed. Results showed that 3-hydroxy-5-methoxy bibenzyl and 3′-*O*-methylbatatasin III were below their active concentrations. However, both compounds coelonin (C1, 4.76 *μ*g/mL) and batatasin III (C2, 9.75 *μ*g/mL) showed remarkable inhibitory effect on all cytokines at this concentration, in particular, the former demonstrated the same TNF-*α* inhibition rate as MC1-4 (Figure 10(d)). The results indicated that for all compounds to show their anti-inflammatory activity, a higher concentration should be applied. Additionally, attention should be paid to the possible synergistic interaction among compounds in the future research.

We found that Wang et al. [43] also reported an anti-inflammatory, antioxidant system regulating an active part of *Bletilla striata*. Briefly, the 95% ethanol extract of *Bletilla striata* was fractionated by systematic solvent; then, the ethyl acetate fraction was eluted by gradient ethanol on MCI column chromatography, combined with total phenol content and DPPH free radical scavenging activity screening; an active fraction BM60 was obtained and characterized by HPLC-ESI-HRMS. Interestingly, the active compounds coelonin and batatasin III also exist in BM60, and EFBS showed similar anti-inflammatory effects to BM60; however, the active dosage of EFBS prepared by us was significantly lower than that of BM60 *in vivo*. This difference is due to the lack of compounds 3′-O-methylbatatasin III and 3-hydroxy-5-methoxy bibenzyl in BM60, or to the difference of other substance combinations or experimental animals, which needs further study. However, it is obvious that the preparation method of EFBS is much simpler than that of BM60. More importantly, the quantitative analysis of the main active components of EFBS was also carried out, and the pharmacodynamic contribution and possible molecular mechanism of the main components were further verified by the component-knockout technique. Therefore, this work is still innovative and instructive for the future development and utilization of *Bletilla striata*.

## 5. Conclusions

In conclusion, all the results presented herein confirm that the standardized EFBS of *Bletilla striata* has remarkable anti-inflammatory effects on LPS-induced ALI by reducing neutrophil infiltration and downregulating inflammatory cytokines expression. Additionally, it is worth noting that this research is the first to quantitatively analyze the extract of *Bletilla striata* and reveal its potential mechanism of action against LPS-induced ALI. Thus, the present study confirms the pharmacological effects of *Bletilla striata* and provides a theoretical and experimental basis for its clinical application and development as a new drug.

## Data Availability

All data generated or analyzed during this study are included in this published article.

## Abbreviations

EFBS: Effect fraction of *Bletilla striata*
KO-EFBS: The four main compounds knockout-EFBS
MC1-4: Mixed-compounds 1-4 fraction
HPLC: High-performance liquid chromatography
CFDA: China food and drug administration
LPS: Lipopolysaccharide
ALI: Acute lung injury
W/D: Wet-to-dry weight ratio
BALF: Bronchoalveolar lavage fluid
iNOS: Inducible nitric oxide synthase
NF-*κ*B: Nuclear factor kappa-B
COX2: Cyclooxygenase-2
JNK: c-Jun N-terminal kinase
ERK: Extracellular-regulated protein kinases
p38: p38 mitogen-activated protein kinase
IL: Interleukin
TNF-*α*: Tumor necrosis factor-*α*
MCP-1: Monocyte chemotactic protein 1
ROSs: Reactive oxygen species
NO: Nitric oxide
MDA: Malondialdehyde
MPO: Myeloperoxidase
DEX: Dexamethasone.
